# Potential chemoprotective effects of active ingredients in *Salvia miltiorrhiza* on doxorubicin-induced cardiotoxicity: a systematic review of *in vitro* and *in vivo* studies

**DOI:** 10.3389/fcvm.2023.1267525

**Published:** 2023-10-17

**Authors:** Qingqing Wang, Jiaxian Li, Xuelei Chu, Xiaochen Jiang, Chuanlong Zhang, Fudong Liu, Xiyuan Zhang, Yi Li, Qian Shen, Bo Pang

**Affiliations:** ^1^ Guang’anmen Hospital, China Academy of Chinese Medical Sciences, Beijing, China; ^2^ Beijing Association of the Integrating of Traditional and Westem Medicine, Beijing, China; ^3^ Eye Hospital, China Academy of Chinese Medical Sciences, Beijing, China; ^4^ Wangjing Hospital, China Academy of Chinese Medical Sciences, Beijing, China; ^5^ Graduate School of Beijing University of Chinese Medicine, Beijing, China

**Keywords:** *Salvia miltiorrhiza*, doxorubicin, Adriamycin, cardiotoxicity, cardioprotection

## Abstract

**Background:**

Recently, attention has been paid to the protective properties of active ingredients in *Salvia miltiorrhiza* (AISM) against organ toxicity induced by chemotherapy drugs. Purpose of the present systematic review is to evaluate the chemoprotective effects and mechanisms of AISM on *in vitro and in vivo* models of doxorubicin-induced cardiotoxicity (DIC).

**Methods:**

According to the PRISMA guideline, the current systematic review was conducted in the Web of Science, PubMed, Embase, and the Cochrane Library to collect all relevant *in vitro and in vivo* studies on “the role of AISM on DIC” published up until May 2023. The SYRCLE's tool was used to identify potential risk of bias.

**Results:**

Twenty-two eligible articles were included in this systematic review. Eleven types of active ingredients in *Salvia miltiorrhiza* were used for DIC, which have the following effects: improvement of physical signs and biochemical indicators, reduction of cardiac function damage caused by DIC, protection of heart tissue structure, enhancement of myocardial cell viability, prevention of cardiomyocyte apoptosis, increase of the chemosensitivity of cancer cells to Doxorubicin, *etc*. The cardioprotective mechanism of AISM involves inhibiting apoptosis, attenuating oxidative stress, suppressing endoplasmic reticulum (ER) stress, decreasing inflammation, improving mitochondrial structure and function, affecting cellular autophagy and calcium homeostasis. The quality scores of included studies ranged from 4 to 7 points (a total of 10 points), according to SYRCLE's risk of bias tool.

**Conclusion:**

This systematic review demonstrated that AISM have chemoprotective effects on DIC *in vivo* and *in vitro* models through several main mechanisms such as anti-apoptosis, antioxidant effects, anti-ER stress, and anti-inflammatory.

## 1. Introduction

Cancer is the main cause of death and an important obstacle to extending life expectancy in countries around the world (1). Worldwide, an estimated 19.3 million new cancer cases and almost 10.0 million cancer deaths occurred in 2020. The global cancer burden is expected to be 28.4 million cases in 2040, a 47% rise from 2020. Overall, the burden of cancer incidence and mortality is rapidly growing worldwide (2). The conventional therapies for cancer are surgery, radiotherapy, and chemotherapy (3). Although chemotherapy is effectively used for systemic treatment of different cancers, its clinical application is limited by some shortcomings, such as the lack of tumor selectivity, which leads to serious toxic side effects on normal tissues and organs (4–7).

Adriamycin (Doxorubicin, DOX) is an anthracycline antibiotic, produced by *Streptomyces peucetius* bacteria and was first used as a cytotoxic medication in 1969 (8). Due to its excellent anti-tumor properties, it has always been used as one of the most commonly used and effective anti-tumor drugs alone or in combination with other drugs (9). When DOX is given as a single drug or in combination with other anti-tumor drugs, the most common tumor reactions include breast cancer and esophageal cancer; osteosarcoma, Kaposi's sarcoma, and soft tissue sarcoma; And Hodgkin's lymphoma and non-Hodgkin's lymphoma. Other cancers that have poor response to doxorubicin but can still be treated with the drug due to its overall benefits include gastric cancer, liver cancer, bile duct cancer, pancreatic cancer and endometrial cancer (10). DOX has pleiotropic anticancer activity, including its contribution to DNA damage, reactive oxygen species (ROS) production, apoptosis, senescence, autophagy, ferroptosis, and pyroptosis induction, as well as its immunomodulatory role (11). However, the harmful effects of DOX are not unique to cancer cells, as it affects both healthy and cancer cells, leading to multiple organ damage (12). Dose dependent cardiotoxicity is considered the most relevant side effect of DOX. DOX at doses of 500–550 mg/m^2^ causes approximately 4% of patients to develop cardiomyopathy, while at doses of 551–600 mg/m^2^, it is approximately 18%. Over 600 mg/m^2^ causes 36% of patients to develop cardiomyopathy (13). This cardiac toxicity can be classified as acute (dose dependent) or chronic (cumulative) (14, 15). Acute cardiac toxicity is characterized by chest pain, tachycardia, and other electrocardiogram changes (16). Chronic cardiotoxicity is permanent and irreversible (17). The most serious chronic side effect of the heart is dilated cardiomyopathy, which may develop 10–15 years after treatment and lead to congestive heart failure directly related to the cumulative dose of DOX (18, 19). In addition to the heart, DOX also induced changes in organs such as the liver, kidney, testicle, and brain (20–23). Given the dual nature of DOX, it is recommended to use chemical protectants during doxorubicin treatment, which may reduce adverse reactions and improve patient survival.

In the past few decades, the use of herbs and natural products or their derivatives to reduce adverse reactions caused by chemotherapy or increase the sensitivity of cancer cells to chemotherapy drugs has attracted widespread attention. *Salvia miltiorrhiza* (Danshen, Simplified Chinese: 丹参) is a common medicinal herb, first recorded in the oldest medical monograph in China, Shennong's Classic of Materia Medica (Shennong Bencao Jing). Its roots have high medicinal value in traditional Chinese medicine, and has been used as a medicated diet in Asia for thousands of years (24). As one of the most commonly used traditional drugs, Danshen has been used to treat various diseases, including cardiovascular disease, cerebrovascular disease, neurodegenerative disease, diabetes, *etc* (25–30). Until now, more than 200 natural compounds extracted from *Salvia miltiorrhiza* have been identified, collectively known as active ingredients in *Salvia miltiorrhiza* (AISM), mainly including lipophilic diterpenoids, such as tanshinone I (Tan I), tanshinone IIA (Tan IIA), tanshinone IIB (Tan IIB), cryptotanshinone (CPT), dihydrotanshinone I (DHT), *etc.*, water-soluble phenolic acids, such as danshensu (DSS), salvianolic acid A and B (Sai A and Sai B), protocatechuic aldehyde, *etc.*, and other constituents, which have exhibited various pharmacological activities, such as anti-inflammation, anti-oxidation, anti-atherogenesis, and anti-diabetes (31). Importantly, these compounds have the ability to kill tumor cells and make tumor cells more sensitive to treatment methods such as chemotherapy and radiation therapy (32). Zhang W et al. reported that Tan IIA significantly inhibited the proliferation of several types of tumors, blocked the cell cycle, induced apoptosis and autophagic death, in addition to inhibiting cell migration and invasion (33). Cai Zhang et al. found that salvianolic acid increased the accumulation of doxorubicin in brain tumors through caveolae endocytosis (34). In addition, their role as chemosensitizers has also been verified in cancer cells such as breast cancer cells and ovarian cancer cells (35, 36). Recently, attention has been paid to the other biological activities of *Salvia miltiorrhiza*, including its protective properties against organ toxicity induced by chemotherapy drugs. Li K et al. reported that Tan IIA enhanced Dox's chemotherapeutic effect on breast cancer, while reducing its side effects, including weight loss, bone marrow suppression, cardiotoxicity and nephrotoxicity (37). Wenjing Ma et al. found that salvianolic acid C effectively reduced the risk of drug-induced immune thrombocytopenia and venous thromboembolism induced by Dox and the repercussions of amplified platelet-cancer interaction in the tumor microenvironment (38). Other studies also found that Tan IIA and Sai A have antagonism on DOX induced nephrotoxicity (39, 40).

Now, focusing on the heart, the current study aimed to evaluate the chemoprotective effects of active ingredients in *Salvia miltiorrhiza* on *in vitro and in vivo* models of doxorubicin-induced cardiotoxicity through a systematic review of the literature, to provide more possible options for developing ideal cardioprotective agents.

## 2. Methods

The Preferred Reporting Items for PRISMA was used to design the current systematic review (41). At each stage of the study, including study search and selection, data extraction, and risk of bias assessment, two independent researchers participated.

### 2.1. Searching strategy

Searches were conducted on the Web of Science, PubMed, Embase, and the Cochrane Library. Two authors independently searched all original articles that had been published up until May, 2023. Due to a linguistic constraint for the selection, only articles in the English language were taken into account. The following diseases and treatments were identified using a combination of MeSH and free text terms:

i) *Salvia miltiorrhiza* or Danshen extract or tanshinone or Cryptotanshinone or Danshensu or Salvianolic acid

and

ii) Doxorubicin

All the articles from these searches were exported to EndNote X8, duplicate records, reviews, and conference abstracts were deleted. First, articles were screened by reading their titles and abstracts; those that were unrelated or lacking full text were then disqualified. The remaining articles were then assessed using the inclusion and exclusion criteria after being read in their entirety.

### 2.2. Inclusion and exclusion criteria

The inclusion criteria were taken into account: (1) *in vitro* and *in vivo* studies; (2) Studies that focused on active ingredients in *Salvia miltiorrhiza* vs. doxorubicin-induced cardiotoxicity; (3) Original data that is independent and full-text searchable.

The exclusion criteria were taken into account: (1) Studies focused on the active ingredients extracted from non-*Salvia miltiorrhiza* plants; (2) Studies focused on organ toxicity other than cardiotoxicity; (3) Research on combination with other drugs; (4) Lack of control.

### 2.3. Data extraction and management

A Microsoft Excel sheet was used by two researchers to separately collect data. When a consensus could not be reached about a discrepancy, the third reviewer was consulted. The following details were taken out of each study: (1) author, year of publication; (2) models (*in vivo*, or/and *in vitro*); (3) doxorubicin dosage, usage, and administration route; (4) outcomes of doxorubicin on cardiac cells/tissue; (5) types of *Salvia miltiorrhiza* extract, dosage, usage, and administration route; (6) outcomes after active ingredients in *Salvia miltiorrhiza* coadministration; (7) the major findings of each article.

### 2.4. Methodological quality appraisal for *in vivo* studies

The methodological quality of *in vivo* studies was assessed using the SYRCLE's risk of bias (RoB) tool (42). It consists of ten items within six main domains. For the judgment of bias, the response options were “YES” to indicate a low risk of prejudice, “NO” to indicate a high risk of bias, or “NC” (NOT CLEAR) to indicate an undetermined level of bias due to insufficient data. The items judged as “YES” were scored one point, and the scores of 10 items were added together for the quality score of each study.

## 3. Results

### 3.1. Study inclusion

**Figure 1:**
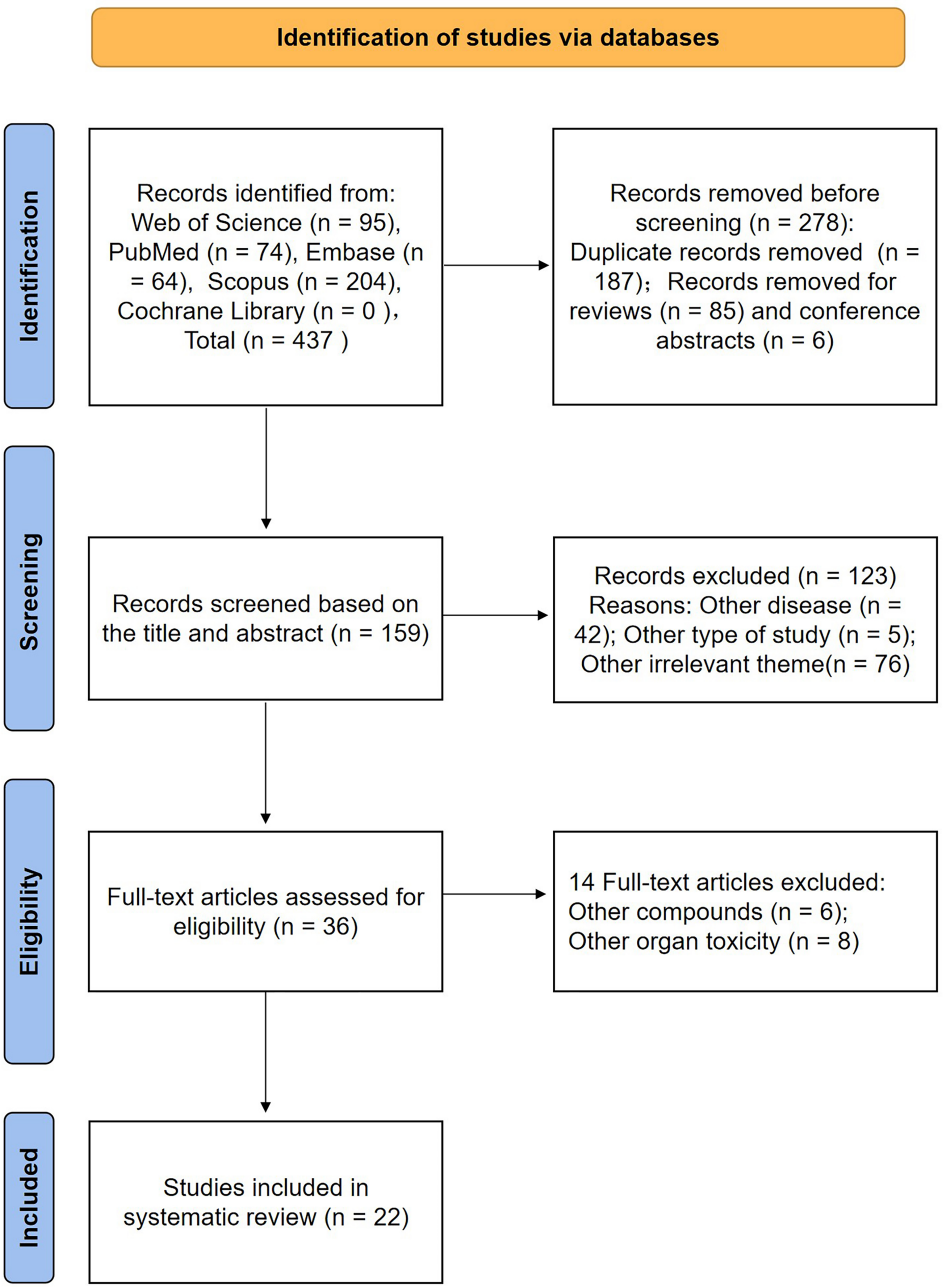
Flow chart of the results according to the search strategies.

437 articles in total, of which 95 appeared in Web of Science, 74 in PubMed, 64 in Embase, and 204 in Scopus (Nothing was found in the Cochrane Library), were extracted from the original retrieval. After then, search filters were used to exclude 278 items (187 duplicates, 85 reviews, and 6 conference abstracts). By reading the titles and abstracts, 42 studies focused on other diseases, 5 other types of studies, and 76 other irrelevant studies were excluded. Thus, 36 articles were read in their full text, 14 of these articles were found to have failed at least one criterion (other compounds, other organ toxicity) and were eliminated after analysis. Finally, 22 articles were included in the systematic review (43–64). The process and results were summarized in Figure 1.

### 3.2. Study characteristics

**Figure 2:**
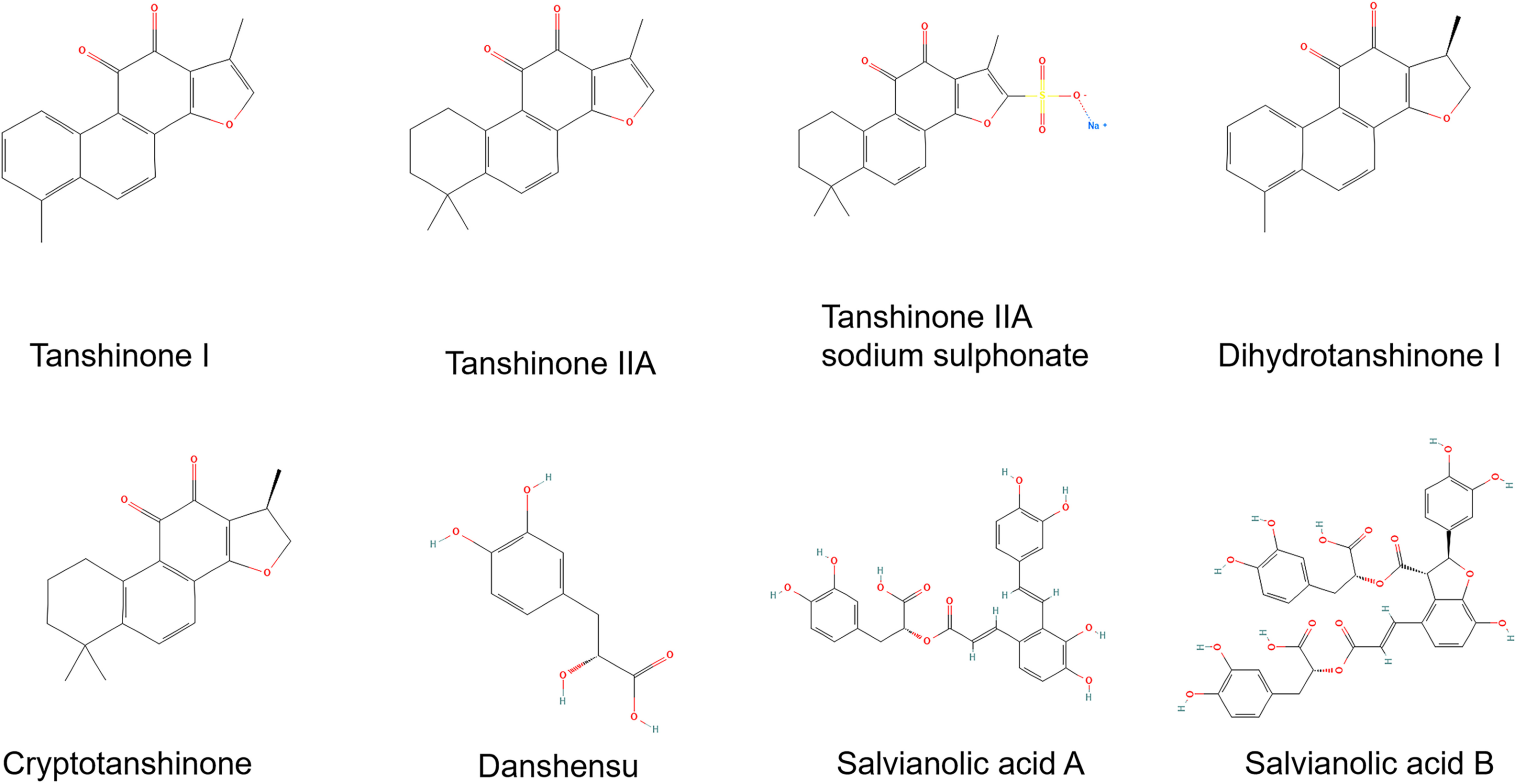
Chemical structures of partial active ingredients in *Salvia miltiorrhiza*.

In the current systematic review, 22 studies were finally included, they contained *in vivo* (5 studies), *in vitro* (6 studies) experiments, and 11 studies done both. Doxorubicin was used to induce cardiac toxicity. The most commonly used concentration for *in vitro* experiments was 1 µm (11 studies), with a minimum of 0.5 µm and a maximum of 200 µm. The dosages of doxorubicin used for *in vivo* studies in different animals are as follows: 3–18 mg/kg i.p. or 4–20 mg/kg i.v. for mice; 1.25–3 mg/kg i.p. for rats. Eleven types of active ingredients in *Salvia miltiorrhiza* were used for doxorubicin-induced cardiotoxicity (DIC), including Tanshinone I (1 studiy; 5, 10 mg/kg, p.o. for *in vivo* and 10µm for mice), Dihydrotanshinone I (1 studiy; 20 mg/kg p.o. for mice and 10 µm for *in vitro*), Tanshinone IIA (6 studies; 2.5–30 mg/kg i.p. or 10 mg/kg p.o. for mice and 0.1–40 µm for *in vitro*), Tanshinone IIA sodium sulphonate (TSNIIA-SS; 2 studies; 30 mg/kg i.p. for mice and 1.6 µm-0.5 mm for *in vitro*), Salvianolic acids (SA; 1 studiy; 40 mg/kg, i.p. for mice), Salvianolic acid A (2 studies; 50 mg/kg i.p. for mice and 2 µm-1 mm for *in vitro*), Salvianolic acid B (2 studies; 2 mg/kg i.p. for mice or 0.25–1 mg/kg i.v. for rats and 20 µg/ml for *in vitro*), Cryptotanshinone (3 studies; 50 mg/kg, p.o. for rats and 2–25 µm for *in vitro*), Danshensu (1 studiy; 50, 100 mg/kg, i.p. for mice), Diethyl Blechnic (DB; 1 studiy; 5–20 μm for *in vitro*), *Salvia miltiorrhiza* aqueous extract (SMAE; 2 studies; 20–100 mg/kg, p.o. for rats and 0.3125–10 mg/ml for *in vitro*). Some of compounds's chemical structures were shown in Figure 2. The details of the study characteristics and the effects of active ingredients in *Salvia miltiorrhiza* on doxorubicin-induced cardiotoxicity were shown in Table 1.

**Table 1:** Chemoprotective effects of active ingredients in *Salvia miltiorrhiza* on doxorubicin-induced cardiotoxicity.

Author, year	Model	DOX dosage, usage, and administration route	Outcomes of DOX on cardiac cells/tissue	Types of AISM	AISM dosage, usage, and administration route	Outcomes of AISM coadministration	Major findings
Lin et al. (43)	*In vitro*/mitochondria from rat heart, and *in vivo*/mice with P388 ascites tumor	DOX 50, 100, and 200 μm + Fe^2+^ 15 μm, 1 h (for *in vitro*); and 5 mg/kg, i.p., once (for *in vivo*)	↑ Lipid peroxidation (↑ MDA levels); ↑ membrane rigidification on mitochondria from rat heart; ↑ production of highly reactive hydroxyl radicals	Salvianolic acid A	1 mm, 1 h (for *in vitro*/mitochondria) and 50 mg/kg, i.p., once daily, 7 days (for *in vivo*)	↓ MDA levels; inhibited rigidification of mitochondrial membrane; scavenged hydroxyl radicals; antitumor action of DOX was not antagonized by Sai A	Sai A protects against adriamycin induced heart mitochondrial toxicity of rats, while it has no antagonizing effect on the antitumor activity of DOX.
Zhou et al. (44)	*In vivo*/BALB/c mice, and *in vitro*/mitochondria from mouse heart	4 mg/kg, i.v., once weekly, 4 weeks (for *in vivo*); and DOX + Fe^2+^ 50 μm, 1 h (for *in vitro*)	↓ body weight; ↑ lipid peroxidation (↑ myocardial TBARS content); ↑ mitochondrial swelling; ↑ semiquinone radical	Tanshinone IIA sodium sulphonate	30 mg/kg, i.p., once weekly, 4 weeks (for *in vivo*) and 0.05, 0.1, 0.2, 0.5 mm, 1 h (for *in vitro*)	↑ Body weight; ↓ lipid peroxidation; ↑ total SOD activity; ↓ mitochondrial swelling; scavenged semiquinone free radicals	Protective effects of TSNIIA-SS may not only be related to its antioxidant activity but also to its regulation of antioxidant enzyme activities in the heart.
You et al. (45)	*In vivo*/Wistar rats	3 mg/kg, i.p., three times weekly, for 2 weeks	↑ Enlarged abdomen and ascites; ↑ mortality rate; ↓ body weight, heart weight and ratio of heart to body weight; ↑ cardiomyopathic changes and congestive heart failure; ↓ heart cell DNA, RNA and protein synthesis; cell vacuolization, myofibril loss and disarrangement; ↓ GSH-Px, SOD; ↑ MDA	*Salvia miltiorrhiza* aqueous extract	20, 100 mg/kg, p.o., for 30 days	↓ Ascites; ↓ mortality rate; ↑ body weight, heart weight; ↑ cardiac function; promoted heart cell macromolecular biosynthesis; preserve of myocardial ultrastructure in rats; ↑ GSH-Px, SOD; ↓ MDA	SMAE alleviates DIC through antioxidant stress.
Gao et al. (46)	*In vitro*/neonatal SD rat cardiomyocytes	1 μm for 24 h	↓ Cell viability; ↑ apoptotic cell death; ↑ ROS production; ↓ anti-apoptotic Bcl-2 protein; ↑ pro-apoptotic Bax protein; ↓ Bcl-2/Bax	Tanshinone IIA	0.5–2 μm for 2 h	↑ Cell viability; ↓ apoptotic cell death; ↓ ROS production; ↑ Bcl-2/Bax proteins	Tan IIA inhibits adriamycin-induced cardiomyocyte apoptosis in a dose-dependent manner, and this effect is caused by its antioxidant properties.
Jiang et al. (47)	*In vivo*/KM mice	15 mg/kg, i.p., once	↓ Oxygen radicals absorbance capacities; ↑ MDA; ↓ heart size and body weight; ↓ HW/TL ratios; ↑ CK; induction of histological changes: the cytoplasmic vacuole formation and myofibrillar loss in heart; ↑ ST-interval of ECG	Salvianolic acids	40 mg/kg, i.p., once daily, for 3 connective days	↑ Oxygen radicals absorbance capacities; ↓ MDA; ↑ body weight; ↑ HW/TL ratios; ↓ CK; reduction of histological heart myocardial lesions; reduction of the ST-interval on ECG	SA on cardioprotection through blocking oxidative stress.
Jiang et al. (48)	*In vitro*/H9c2 cells, and *in vivo*/KM mice	1 μm for 24 h (for *in vitro*); and 5 mg/kg, i.p., once daily at days 1, 8, 15 (for *in vivo*)	↓ Cell viability; ↑ apoptotic cell death; ↓ heart rate; ↑ ST-interval and QRS interval duration of ECG; ↓ myocardial tensile strength; ↓ heart size and body weight; ↓ HW/TL ratio; induction of histological changes: the cytoplasmic vacuole formation and myofibrillar loss in heart, fibrosis around the arterioles	Tanshinone IIA sodium sulfonate	1.6, 8, 40 μm for 24 h (for *in vitro*) and TSNIIA dosage not clear i.p., once daily, at days 1–3, 8–9, 15–17(for *in vivo*)	↑ Cell viability; ↓ apoptotic cell death; ↑ heart rate; reduction of the ST-interval and QRS interval duration on ECG; ↑ myocardial tensile strength; ↑ heart size; ↑ HW/TL ratio; reduction of histological heart myocardial lesions	TSNIIA-SS exerts a protective effect against DIC by improving the structure and function of myocardial cells.
Hong et al. (49)	*In vitro*/neonatal SD rat cardiomyocytes	1 μm for 24 h	↑ Apoptotic cell death; ↑ caspase 3 activity; ↑ cytosol cytochrome c protein; ↑ ROS production; ↓ Bcl-x_L_ protein; ↓ Akt phosphorylation	Tanshinone IIA	0.1, 0.3, 1, 3μ*Μ* for 0.5 h	↓ Apoptotic cell death; ↓ caspase 3 expression; ↓ cytosol cytochrome c protein; ↓ ROS production; ↑ Bcl-x_L_ protein; ↑ Akt phosphorylation	Tan IIA protects cardiomyocytes from doxorubicin-induced apoptosis through Akt-signaling pathways.
Zhang et al. (51)	*In vivo*/Wistar rats	1.25 mg/kg, i.p., every 2 days, six times for a total of 12 days (2 days 6 doses)	↓ The activities of mitochondrial complexes I, II, III, and IV; ↓ ATP generation; ↓ MMP; ↑ the release of superoxide anion free radical; ↓ the gene and protein level of mitochondrial biogenesis-relative factors PGC-1α, NRF-1, and TFAM; ↑ NO and iNOS; ↓ GSH-Px	Cryptotanshinone	50 mg/kg, p.o., for a total of 20 days	↑ The activities of mitochondrial complexes I, III, and IV; ↑ ATP generation; ↑ MMP; ↓ the release of superoxide anion free radical; ↑ the gene and protein level of mitochondrial biogenesis-relative factors PGC-1α, NRF-1, and TFAM; ↓ NO and iNOS; ↑ GSH-Px	CPT protects against DOX induced mitochondrial dysfunction in cardiomyocytes by increasing the ATP generation, up-regulating the expressions of mitochondrial biogenesis-relative genes, and antioxidant stress.
Chen et al. (50)	*In vivo*/BALB/c mice	20 mg/kg, i.v., once	↓ Heart and body weight; ↓ EF and FS; ↑ LVIDd and LVIDs; ↑ the serum levels of LDH, CK and AST; induction of histological changes: cytoplasmic vacuolisation, myofibrillar loss, mitochondrial oedema, chromatin condensation and cardiomyocyte necrosis; ↓ heart rate; ↑ apoptotic cell death; ↑ cleaved caspase-3, caspase-12; ↓ Bcl-2/Bax ratio; ↑ ER stress markers: GRP78 and CHOP proteins; ↑ proteins expression of p-IRE-1, P-JNK, ATF-6 and p-PERK; ↓ phosphorylation of Akt and GSK3β	Salvianolic acid B	2 mg/kg, i.p., every day for one week	↑ Heart and body weight; ↑ EF and FS; ↓ LVIDd and LVIDs; ↑ LDH, CK, and AST; partially prevented structural abnormalities of heart tissues; ↑ heart rate; ↓ apoptotic cell death; ↓ cleaved caspase-3, caspase-12; ↑ Bcl-2/Bax ratio; ↓ ER stress markers: GRP78 and CHOP proteins; ↓ proteins expression of p-IRE-1, P-JNK, ATF-6 and p-PERK; ↑ phospho-Akt and phospho-GSK3β	Sai B protects against DIC by inhibiting endoplasmic reticulum stress, and by being involved in the PI3K/Akt pathway.
Chen et al. (52)	*In vivo*/SD rats, and *in vitro*/rat ventricular myocytes	3 mg/kg, i.p., every 2 days for three injections (for *in vivo*); and 1 µm for 4 h (for *in vitro*)	↓ Heart and body weight; ↑ LDH level; induction of histological changes: cytoplasmic vacuolisation, myofibrillar loss, mitochondrial oedema, chromatin condensation and cardiomyocyte necrosis; ↑ apoptotic cell death; ↓ cardiomyocyte contractility; ↓ Bcl-2 protein; ↑ proteins expression of Bax, cleaved caspase-3, GRP78, and CHOP; ↑ TRPC3 and TRPC6	Salvianolic acid B	0.25, 0.5, 1 mg/kg i.v., for 7 days (for *in vivo*) and 20 µg/ml for 6 h (for *in vitro*)	↑ Heart and body weight; ↓ LDH level; partially prevented structural abnormalities of heart tissues; ↓ apoptotic cell death; ↑ cardiomyocyte contractility; ↑ Bcl-2 protein; ↓ proteins expression of Bax protein, cleaved caspase-3, GRP78, and CHOP; ↓ TRPC3 and TRPC6	Sai B protects against DOX-induced cardiac apoptosis and ER stress via TRPC3 and TRPC6 inhibition.
Song et al. (53)	*In vitro*/rat myocardial cells H9c2	10, 50 μm for 24 h	↓ Cell growth and relative viability; ↓ cell apoptosis; ↓ miR-133; ↑ expression of Caspase-9, cleaved Caspase-3, and cleaved PARP	Tanshinone IIA	5, 10, 15, 20, 25, 30 μm for 24 h	↓ Apoptotic cell death; ↑ cell growth and viability; ↑ miR-133; ↓ expression of Caspase-9, cleaved Caspase-3, and cleaved PARP	Tan ⅡA ameliorated myocardial apoptosis via restoration of miR-133 and suppression Caspase-9 signaling cascade.
Guo et al. (54)	*In vivo*/mice, and *in vitro*/H9c2 rat myoblast cell line	18 mg/kg, i.p., once (for *in vivo*); and 1 µm for 24 h (for *in vitro*)	Induction of histological changes: myocardial fiber fragmentation and gap enlargement; ↑ serum myocardial enzymes (AST, LDH, CK and CK-MB); ↓ SOD and CAT activities, GSH content; ↑ MDA production; ↑ mRNA levels of NQO1, MRP2, and P-gp; ↓ cell viability; ↑ ROS production	Tanshinone IIA	15 and 30 mg/kg i.p., for 7 days (for *in vivo*) and 1, 3, 5 and 10 µm for 4 h (for *in vitro*)	Prevented structural abnormalities of heart tissues; ↓ AST, LDH and CK (30 mg/kg); ↓ CK-MB activity (15, 30 mg/kg); ↑ SOD and CAT activities, GSH content; ↓ MDA production; ↑ Nrf2, HO-1, NQO1, and GCLC; ↓ MRP2 and P-gp; ↑ cell viability (1–10 µm); ↓ ROS production	The Nrf2-dependent antioxidant response mediates the protective effect of Tan IIA on DIC.
Yu et al. (55)	*In vitro*/H9c2 cells and rat neonatal cardiomyocytes	1 μm for 24 h	↓ Cell viability; ↑ apoptotic cell death; ↓ MMP; ↓ Bcl-2 and Bcl-xl proteins, ↑ Bax protein; ↑ proteins levels of p-p53 and cyt c; ↓ survivin; ↑ cleaved caspase 3, 7, 8, 9 protein levels, and the activities of caspase 3/7; ↑ ROS; ↑ phosphorylated of ERK1/2, JNK1/2, and p38	Diethyl Blechnic	5, 10, 20 μm for 2, 24 h	↓ Apoptotic cell death; ↑ cell viability; ↑ MMP; ↑ Bcl-2 and Bcl-xl proteins, ↓ Bax protein; ↓ proteins levels of p-p53 and cyt c; ↑ survivin; ↓ cleaved caspase 3, 7, 8, 9 protein levels, and the activities of caspase 3/7; ↓ ROS; ↑ phosphorylated of ERK1/2, JNK1/2, and p38	DB protects cardiomyocytes against DOX-induced cytotoxicity by inhibiting ROS and activating the JNK1/2 pathway.
Wang et al. (56)	*In vivo*/zebrafish, C57BL/6 mice, and *in vitro*/H9C2 cells, U87 cells	100 µm (for zebrafish); 5 mg/kg, i.v., once per week, 4 consecutive weeks (for mice); 1 µm for 24 h (for H9C2 cells); and 1 µm for 24 h/48 h/72 h (for U87 cells)	↓ EF and FS values of echocardiographs; ↑ LVEDD and LVESD; induction of histological changes: the disorderly arrangement of cardiac tissue, myofibrillar loss; ↑ LDH and CK-MB; ↑ apoptotic cell death; ↓ body weight; ↓ Bcl-2 protein; ↑ Bax protein; ↑ accumulation of autolysosomes; ↓ cathepsin B activity; ↑ LC3-II and P62; ↓ Beclin1 and LAMP1; ↑ Ser2448p-mTOR; ↑ Ser757p-ULK1; ↑ Thr389P-S6K; ↓ TFEB	Tanshinone IIA	20 µm (for zebrafish);10 mg/kg p.o., 4 weeks (for mice); 2 µm for 24 h (for H9C2 cells); and 1, 5, and 20 µm for 24/48/72 h (for U87 cells)	↑ EF and FS values of echocardiographs; ↓ LVEDD and LVESD; prevented structural abnormalities of heart tissues; ↓ LDH and CK-MB; ↓ apoptotic cell death; ↑ body weight; ↑ Bcl-2 protein; ↓ Bax protein; ↓ accumulation of autolysosomes; ↑ cathepsin B activity; ↓ LC3-II and P62; ↑ Beclin1 and LAMP1; ↓ Ser2448p-mTOR; ↓ Ser757p-ULK1; ↓Thr389P-S6K; ↑ TFEB	Tan IIA protects against DIC by promoting autophagy via the Beclin1/LAMP1 signaling pathway, and it is able to reduce the cardiotoxicity of DOX without compromising antitumor activity.
Hung et al. (57)	*In vivo*/Wistar rats, and *in vitro*/H9c2 cells	Six equal doses (each containing 3 mg/kg) over a period of 2 weeks, i.p., (for *in vivo*); and 1 µm for 24 h (for *in vitro*)	Induction of histological changes: collagen accumulation; ↑ apoptotic cell death; ↑ caspase-3; ↑ the extent of protein oxidation; ↓ SOD production; ↑ ROS production; ↓ Nrf2 and HO-1 proteins expression; ↑ phosphorylated ERK1/2 and p53 protein; ↑ cleaved PARP; ↑ cathepsin B; ↓ AIF	*Salvia miltiorrhiza* aqueous extract	100 mg/kg/day, p.o., for 5 weeks (for *in vivo*) and 0.3125, 0.625, 1.25, 2.5, 5, and 10 mg/ml of SMAE for 48 h (for *in vitro*)	Prevented structural abnormalities of heart tissues; ↓ apoptotic cell death; ↓ caspase-3; ↓ protein carbonylation; ↑ SOD production; ↓ ROS production; ↑ Nrf2 and HO-1 proteins expression; ↓ phosphorylated ERK1/2 and p53 protein; ↓ cleaved PARP; ↓ cathepsin B; ↑ AIF	ROS apoptosis-inducing molecule release is closely involved in DIC while SMAE could prevent or mitigate the causative cardiomyopathy through controlling multiple targets without compromising the efficacy of chemotherapy.
Li et al. (58)	*In vivo*/Wistar rats, and *in vitro*/H9c2 cells	2 mg/kg, i.p., with three injections each week and a total of six injections (for *in vivo*); and 2 µm for 3, 6, 12 h (for *in vitro*)	↓ Cardiomyocyte viability; ↓ cell surface area; ↑ apoptotic cell death; ↓ MMP; ↑ ROS; ↓ cardiac dysfunction: EF and FS values; induction of histological changes: nuclear cavity, cardiomyocytes loosely aligned, cell surface area reduced; ↓ SOD, CAT, and GSH-Px; ↑ MDA; ↑ 14–3-3σ and JNK; ↓ PI3 kinase p85 and p-AKT; ↓ Bcl-2 and Bcl-xl; ↑ Bax, Bak, Bim, and PUMA; ↑ cleaved caspase-9 and caspase-3; the nuclear translocation of p53 and Foxo1	Cryptotanshinone	50 mg/kg, p.o., once every 2 days for 6 weeks (for *in vivo*) and 2, 5, 10 µm for 3, 6, 12 h (for *in vitro*)	↑ Cell viability (10 µm); ↑ cell surface area; ↓ apoptotic cell death; ↑ MMP; ↓ ROS; ↑ EF and FS; prevented structural abnormalities of heart tissues; ↑ SOD, CAT, and GSH-Px; ↓ MDA; ↓ 14-3-3σ and JNK; ↑ PI3 kinase p85 and p-AKT; ↑ Bcl-2 and Bcl-xl; ↓ Bax, Bak, Bim, and PUMA; ↓ cleaved caspase-9 and caspase-3; suppressed p53 nuclear translocation and enhanced Foxo1 nuclear retention	CPT suppresses DIC by inhibiting p53 signaling pathway.
Wang et al. (59)	*In vivo*/zebrafish, C57BL/6 mice, and *in vitro*/ H9C2 cells	100 µm (for zebrafish); 5 mg/kg, i.v., once per week, 4 consecutive weeks (for mice); 1 µm for 24 h (for H9C2 cells)	↓ FS, erythrocyte circulation within tail blood vessels, heart rate and survival rate in DIC zebrafish; ↑ LVEDD and LVESD; ↓ FS and EF; ↑ MDA; ↓ SOD; ↑ the percentage of macrophages; ↑ M1 macrophages; ↑ the protein expressions of CD86 and F4/80; ↑ p-NF-κB, TNF-α, COX2, and IL-8; ↑ levels of phosphorylated mTOR and S6K; ↑ cleaved caspase 3 and Bax; ↓ Bcl-2	Dihydrotanshinone I	10 nM (for zebrafish); 20 mg/kg p.o., 4 weeks (for mice); 10 nM for 24 h (for H9C2 cells)	↑ FS, erythrocyte circulation within tail blood vessels, heart rate and survival rate;↑ EF and FS values; ↓ LVEDD and LVESD; ↓ MDA; ↑ SOD; ↓ recruitment of macrophages; ↓ M1 macrophages; ↓ CD86 and F4/80; ↓ p-NF-κB, TNF-α, COX2, and IL-8; ↓ levels of phosphorylated mTOR and S6K; ↓ cleaved caspase 3 and Bax; ↑ Bcl-2	DHT can be applied as a novel cardioprotective compound in the antiinflammation management of DIC via mTOR-TFEB-NF-κB signaling pathway.
Wang et al. (60)	*In vitro*/H9c2 cells	1 μm for 24 h	↓ Cell viability; ↑ apoptotic cell death; ↑ ROS; ↓ SOD2; ↓ Bcl-2; ↓ the expressions of Bax, cleaved caspase 3, caspase 7; ↓ MMP; ↓ production of ATP; ↓ the levels of p-Akt and p-GSK3β; ↓ the binding of phospho-GSK-3β to ANT; ↓ the formation of the ANT-Cyp-D complex	Cryptotanshinone	5, 10, 25 μm for 24 h	↑ Cell viability; ↓ apoptotic cell death; ↑ Bcl-2; ↓ Bax; ↓ cleaved caspase 3, 7; ↓ ROS; ↑ SOD2; ↑ MMP; ↑ production of ATP; ↑ the levels of p-Akt and p-GSK3β; ↑ the binding of phospho-GSK-3β to ANT; ↑ the formation of the ANT-Cyp-D complex	CPT could ameliorate oxidative stress and apoptosis via the Akt-GSK-3β-mPTP pathway.
Wu et al. (61)	*In vitro*/the rat cardiomyocytes, H9C2 cells	1, 2, 4, 8 μm for 12 h	↓ Cell viability; ↑ apoptotic cell death; ↓ Bcl-2; ↑ cleaved caspase 3, caspase 9; ↑ NF-kB signaling including IkBα, IKKα, IKKβ, and p65; ↑ NFKB1; ↑ Bax	Salvianolic Acid A	2, 10, 50 μm for 12 h	↑ Cell viability; ↓ apoptotic cell death; ↑ Bcl-2; ↓ cleaved caspase 3, caspase 9; ↓ NF-kB signaling including IkBα, IKKα, IKKb, and p65; ↓ NFKB1; ↓ Bax	Sai A exerts a protective effect against Dox-induced H9C2 injury and apoptosis via inhibition of NFKB1 expression, thereby downregulating lncRNA PVT1.
Jiang et al. (62)	*In vivo*/C57BL/6 mice, and *in vitro*/H9c2 cells	5 mg/kg, i.v., once a week for consecutive 4 weeks, (for *in vivo*); and 0.25–2 µm for 24 h (for *in vitro*)	↓ Cardiac dysfunction: EF and FS values; induction of histological changes: disruption of cardiomyocytes, infiltration of inflammatory cells, and expansion of intercellular spaces. ↑ CK-MB and LDH; ↓ Bcl-2; ↑ Bax; ↑ ROS and MDA; ↓ T-SOD and GSH-Px; ↓ p-AKT, Nrf2, HO-1 and NQO1; ↓ Cell viability; ↑ apoptotic cell death; ↓ MMP	Tanshinone I	5, 10 mg/kg, p.o., daily for 4 weeks (for *in vivo*) and 10 µm for 24 h (for *in vitro*)	↑ Cardiac dysfunction: EF and FS values; induction of histological changes: disruption of cardiomyocytes, infiltration of inflammatory cells, and expansion of intercellular spaces. ↓ CK-MB and LDH; ↑ Bcl-2; ↓ Bax; ↓ ROS and MDA; ↑ T-SOD and GSH-Px; ↑ p-AKT, Nrf2, HO- 1 and NQO1; ↑ Cell viability; ↓ apoptotic cell death; ↑ MMP	Tan I attenuates oxidative stress and protected mitochondria through Nrf2 signaling pathway.
Qi et al. (63)	*In vivo*/KM mice	15 mg/kg, i.p., once	↓ Body weight, food consumption, and water consumption; ↑ ascites; ECG changes: ST-segment elevation and prolongation of QTc interval alleviated; histological changes: a large number of necrotic cardiomyocytes and obvious nuclear lysis; ↑ LDH and CK; ↑ ROS content and MDA concentration; ↓ the activities of SOD, CAT, and GPX; ↑ the levels of TNF-α and IL-6; ↑ Bax/Bcl-2; ↑ caspase 3; ↑ Keap1; ↓ Nrf2, HO-1, and NQO1	Danshensu	50,100 mg/kg, i.p., for 3 days	↑ Body weight, food consumption, and water consumption; ↓ ascites; alleviated ST-segment elevation and prolongation of QTc interval; reduction of cardiac injury; ↓ LDH and CK; ↓ ROS content and MDA concentration; ↑ the activities of SOD, CAT, and GSH-Px; ↓ the levels of TNF-α and IL-6; ↓ Bax/Bcl-2; ↓ caspase 3; ↓ Keap1; ↑ Nrf2, HO-1, and NQO1	DSS could effectively exerts anti-oxidative stress, anti-inflammatory and anti-apoptotic therapeutic effects on DIC by regulating the expression of Keap1-Nrf2/NQO1.
Xu et al. (64)	*In vivo*/C57BL/6 mice, and *in vitro*/H9c2 cells and HL-1 cells	3 mg/kg,i.p., once every three days for a total of 7 injections (for *in vivo*); and 60 µm and 1 µm for 24 h (for *in vitro*)	↓ Cell viability; ↑ apoptotic cell death; ↑ cleaved caspase 3; ↓ DAXX; ↓ p-ERK1/2 and p-MEK; ↑ p-P38 and cleaved caspase-8; ECG changes: ↓ LVEF and LVFS; ↑ LVIDs; histological changes: cardiac fiber disruption and nuclear pyknosis	Tanshinone IIA	2.5, 5, 10 mg/kg i.p., daily, for 7 days (for *in vivo*) and 10, 20 and 40 µm for 24 h (for *in vitro*)	↑ Cell viability; ↓ apoptotic cell death; ↓ cleaved caspase 3; ↑ DAXX; ↑ p-ERK1/2 and p-MEK; ↓ p-P38 and cleaved caspase-8; ECG parameters (LVEF, LVFS and LVIDs) were reversed; preserved the structure of myocardial cells	DAXX exerts an important role in DIC and Tan IIA may be a novel agent strategy for DIC treatment via activating the DAXX/MEK/ ERK1/2 pathway.

In the review, the SYRCLE checklist was used to evaluate the risk of bias for 16 included *in vivo* studies. The quality scores ranged from 4 to 7 points. Baseline characteristics, performance bias, blinding items of detection bias, attrition, reporting, and other sources all had low risks of bias. However, there were also many high-risk items that attention should be paid to the detailed reporting of random sequence generation, allocation concealment, random outcome assessment and the use of blinding in the future, which will improve the reliability and rigor of the studies. The bias risk of *in vivo* studies was summarized in Table 2.

**Table 2:** Bias risk of included *in vivo* studies.

Study/Bias	Selection bias			Performance bias		Detection bias		Attrition bias	Reporting bias	Other bias	Quality score (“YES” items)
	Sequence generation	Baseline characteristics	Allocation concealment	Random housing	Blinding	Random outcome assessment	Blinding	Incomplete outcome data	Selective outcome reporting	Other sources of bias	
Lin et al. (43)	NO	YES	NC	NC	NC	NC	YES	YES	YES	YES	4
Zhou et al. (44)	NC	YES	NC	NC	NC	NC	YES	YES	YES	YES	5
You et al. (45)	NC	NC	NC	NC	NC	NC	YES	YES	YES	YES	5
Jiang, et al. (47)	NC	YES	NC	YES	YES	NC	YES	YES	YES	YES	7
Jiang et al. (48)	NC	YES	NC	NC	NC	NC	YES	YES	YES	YES	5
Zhang et al. (51)	NC	YES	NC	YES	YES	NC	YES	YES	YES	YES	7
Chen et al. (50)	NC	YES	NC	YES	YES	NC	YES	YES	YES	YES	7
Chen et al. (52)	NC	YES	NC	NC	NC	NC	YES	YES	YES	YES	5
Guo et al. (54)	NC	YES	NC	YES	YES	NC	YES	YES	YES	YES	7
Wang et al. (56)	NC	YES	NC	YES	YES	NC	YES	YES	YES	YES	7
Hung et al. (57)	NC	YES	NC	NC	NC	NC	YES	YES	YES	YES	5
Li et al. (58)	NC	YES	NC	YES	YES	NC	YES	YES	YES	YES	7
Wang et al. (59)	NC	YES	NC	YES	YES	NC	YES	YES	YES	YES	7
Jiang et al. (62)	NC	YES	NC	NC	NC	NC	YES	YES	YES	YES	5
Qi et al. (63)	NC	YES	NC	YES	YES	NC	YES	YES	YES	YES	7
Xu et al. (64)	NC	YES	NC	NC	NC	NC	YES	YES	YES	YES	5

### 3.3. The role of active ingredients in *Salvia miltiorrhiza* on doxorubicin induced cardiotoxicity

#### 3.3.1. Effect on physical signs change

Seven studies have reported the effect of AISM on doxorubicin induced changes in animal signs, including body weight, heart weight, ascites, heart rates, food and water consumption, mortality rate, *etc*. The results of this study showed that compared with the control group, the body weight and heart weight of mice/rats in the DOX group decreased (44, 45, 47, 48, 52, 63). We observed a decrease in the ratio of animal heart to body weight and the ratio of heart weight to tibial length after treatment with DOX (45, 47, 48, 50). In addition, compared with untreated rats, rats treated with doxorubicin showed a significant accumulation of ascites (45, 63), and the mortality rate was significantly higher than other rats (45, 63). Compared with the group treated with doxorubicin alone, the combination of AISM and doxorubicin significantly increased the body weight, heart weight, heart-to-body weight ratio, and heart weight to tibia length ratio of mice/rats, as well as food consumption and water consumption. In addition, co-treatment with AISM significantly reduced the increase in ascites value in animals treated with doxorubicin and reduced mortality.

#### 3.3.2. Effect on biochemical markers

Seven studies reported the changes in biochemical markers after DOX. Serum myocardial enzymes are the important indexes that reflect the extent of myocardial injury. Creatine kinase (CK) is an important clinical marker of cardiac injury. Lactate dehydrogenase (LDH) and creatine kinase-muscle/brain (CK-MB) are located in the cytoplasm of cardiomyocytes under normal conditions and the release of LDH and CK-MB into the blood is a diagnostic indicator of heart failure. DOX significantly increased the activity of all enzymes compared with controls, indicating cardiotoxicity. Among these compounds, Tan I reduced serum levels of CK-MB and LDH (63), Tan IIA significantly reduced the levels of serum myocardial enzymes, including aspartate transaminase (AST), LDH, CK and CK-MB (54, 56). Baohong Jiang et al. illustrated that SA significantly decreased the level of CK (47). Sai B has been reported to reduce the levels of LDH, CK and AST in mice (50), as well as the level of LDH in rats (52). In addition, DSS was also found to reduce CK and LDH levels, and exhibited a dose-dependent trend (63).

#### 3.3.3. Effect on cardiac function

Ten studies reported that AISM maintains heart function from DOX cardiotoxicity damage. Jyh Sheng You et al. found that SMAE treatment alleviated doxorubicin induced cardiomyopathy and congestive heart failure, improved cardiac function (45). The electrocardiogram showed that the QTc interval of animals in the DOX group was 2.3 folds higher than that of the normal group, accompanied by significant ST-segment elevation. DSS treatment effectively alleviated the prolongation of QTc interval (63). Echocardiography showed that DOX reduced the ejection fraction (EF) and shortening fraction (FS), increased the left ventricular end-diastolic dimension (LVEDD), left ventricular end-systolic dimension (LVESD), left ventricular internal diameter at diastolic phase (LVIDd), and left ventricle internal diameter in systolic phase (LVIDs) values, indicating severe damage to mouse cardiac function. However, salvianolic acid has been shown to partially reverse these functional changes (47, 50). Similarly, DHT I was proved to increase FS value and promote tail venous blood flow in zebrafish model. In mice, DHT I reduced LVEDD and LVESD values. The significant increase in FS and EF values represented a strengthening in cardiac contractility, indicating that DHT I treatment can improve left ventricular function (59). And, Tan I was also observed to increase EF and FS values in a dose-dependent manner (63). TSNIIA-SS significantly reversed the prolongation of ST and QRS intervals induced by DOX (48). After treatment with Tan IIA, EF, FS, and LVIDs values increased, while LVEDD and LVESD levels significantly decreased (56, 64). Hemodynamic examination showed that DOX induced a left shift of the pressure-volume (PV) loop. The EF and FS of rats treated with simultaneous oral administration of CPT increased, and the PV loop shifted to the right along the horizontal axis, indicating that CPT treatment alleviated dox-induced cardiac dysfunction in rats (58).

#### 3.3.4. Effect on cardiac histology

The morphological and histological changes in the heart have been described in detail in 7 studies. Jyh-Sheng You et al. observed that under the microscope, the myocardial structure of rats in the DOX treatment group showed cell vacuolization, myofibril loss and disorder, while the myocardial ultrastructure of rats in the SMAE + DOX group was basically well preserved (45). Baohong Jiang et al. further evaluated DOX induced cardiac toxicity using H&E staining (47). The hearts of the control group showed normal cell distribution and normal myocardial morphology, while the hearts of DOX treated animals showed cytoplasmic vacuoles and myofibril loss, which were typical manifestations of cardiomyopathy induced by DOX. The myocardial lesions in animals treated with salvianolic acid were significantly reduced. Using the transmission electron microscope, Rongchang Chen et al. observed the obvious abnormalities such as cytoplasmic vacuolization, myofibril loss, mitochondrial edema, chromatin condensation and myocardial necrosis in the heart tissue of DOX treated mice (50). The pretreatment with Sai B partially prevented DOX induced cardiac tissue structural abnormalities (52). DIC model group showed pathological changes such as myocardial cell structure disorder, myofibril loss, karyopyknosis and plasma dissolving myocardial cells, while Tan IIA pretreatment also partially alleviated these injuries (54, 56, 64). Another study found that the heart size of DOX treated mice was smaller, the ventricular cavity was dilated smaller, the formation of cytoplasmic vesicles and the loss of myofibril. The pathological changes in the hearts and myocardial cells of animals treated with TSNIIA-SS were significantly delayed (48). Le Li et al. reported that treatment with CPT reversed the decrease in surface area of myocardial cells induced by DOX and the increase in collagen deposition in the heart (58). Xiaoping Wang et al. found that DOX caused damage to myocardial cells, infiltration of inflammatory cells, and expansion of intercellular spaces (59). Tan I protected their structure and alleviated inflammatory cell infiltration and cell damage caused by DOX (63). In addition, DOX caused the destruction of cardiac myocytes and the disturbance of myofibril. However, the structure of most cells remained normal after DSS treatment, with clear horizontal lines, and only a few necrotic cardiac myocytes existed (63).

#### 3.3.5. Effect on myocardial cells

##### 3.3.5.1 Cell viability and survival

Sixteen studies provided the effect of AISM on cardiac cell viability and survival after treatment with DOX. Qianqian Jiang et al. reported that DOX dose-dependent reduction in cell viability, while Tan I pretreatment increased cell viability in a dose-dependent manner (62). At a concentration of 10 µm, Tan I had the most significant protective effect on DIC. Under the treatment of 10 µm and 25 µm concentrations of DHT I, the cell apoptosis rate was reduced by 22.4% and 19.4%, respectively (59).

Tan IIA has a significant protective effect on DOX induced cardiomyocyte apoptosis and cell viability (46, 48, 49, 53, 54, 56, 64). Its individual treatment does not affect normal cell survival. On the contrary, the use of Tan IIA on DOX treated cells can prevent cell death. Jie Gao et al. demonstrated that Tan IIA (0.5, 1, 2 mol/L) inhibited DOX (1 mol/L) induced cell death in a dose-dependent manner (46). In MTT detection, the survival rates of myocardial cells increased to 85.6%, 89.1%, and 95.7%, respectively. Hoechst staining showed that Tan Ⅱ A significantly reduced the number of apoptotic cells in typical nuclear fragmentation induced by DOX, which were 18.0%, 13.8% and 6.4%. When flow cytometry was used to quantify apoptosis, the percentage of cardiomyocyte apoptosis decreased, to 15.6%, 13.3% and 10.0%, respectively.

Sai A and B exhibited significant protective effects on DOX induced cardiomyocyte apoptosis. 10 and 50 μm Sai A restored the vitality of H9C2 cells, and the apoptosis rate of H9C2 cells treated with DOX and Sai A was lower (61). Sai B pretreatment also reduced the proportion of TUNEL positive cells (50, 52). TUNEL detection and Hoechst staining both showed that CPT significantly reduced the apoptosis rate of cardiac myocytes induced by dox in rats, and CPT alone did not cause collagen deposition and apoptosis in the rat heart (58, 60). The same effect was observed in the SAME (57). In addition, Diethyll Blechnic pretreatment prevented dox-induced cell death in a concentration dependent manner in primary cultured mouse cardiomyocytes (55).

##### 3.3.5.2. Mitochondrial structure and function

Six studies focused on the effect of AISM on mitochondrial function after DIC. Sai A (43) and TSNIIA-SS (44) showed protective effects on cardiac mitochondrial damage induced by DOX. DOX induced rigidification of mitochondrial membrane, mitochondria were swollen, the addition of Sai A and TSNIIA-SS could significantly inhibit these changes. This indicated that these compounds have a protective effect on the integrity and function of mitochondrial membranes. CPT (51, 60) exerted myocardial protective effects by restoring mitochondrial dysfunction caused by DOX. DOX led to a decrease in the activity of mitochondrial complexes and inhibition of ATP generation, while CPT enhanced the activity of complexes and promoted ATP generation. CPT also increased the mitochondrial membrane potential (MMP), promoted the structural repair and functional recovery of mitochondrial membrane. Meanwhile, CPT partially protected and promoted mitochondrial biogenesis by regulating the expression of factors related to mitochondrial biogenesis. Two other compounds, DB (55) and Tan I (63), also showed protective effects on mitochondrial abnormalities induced by DOX. Pretreatment of DB attenuated the decrease of mitochondrial membrane potential induced by doxorubicin, which confirmed its protective effect. Tan I inhibited DOX induced cardiotoxicity and alleviated DOX induced damage to mouse heart mitochondria by regulating nuclear factor (erythroid-derived 2)-like 2 (Nrf2) signaling pathway.

#### 3.3.6. Other benefits

The combination of AISM and DOX has demonstrated potential for anti-tumor and cardiac protection in different experimental models, and may play a role by regulating multiple signaling pathways and protein expression. Tong Jun Lin et al. demonstrated that Sai A alone did not exhibit anti-tumor activity (43). However, when combined with DOX, Sai A will not antagonize its anti-tumor effect, and even SMAE can enhance DOX's inhibitory effect on breast cancer cells (57). Tan IIA has also been found to reduce the toxicity of DOX to the heart without affecting its anti-tumor effect. It increased the chemosensitivity of cancer cells to DOX by inhibiting the expression of multiple drug resistance protein 1 and multiple drug resistance related protein 1 (64). Tan IIA was observed to restore autophagic flux and improve the cell viability of DOX-stimulated H9C2 cells via increasing autophagosome formation and autolysosome degradation, the efficacy of improving autophagic flux was shown to be mediated by the Beclin1/lysosomal-associated membrane proteins-1 (LAMP1) pathway (56). In addition, Sal B alleviated DOX induced cardiomyocyte dysfunction and intracellular calcium disorder, and reduced intracellular calcium overload and endoplasmic reticulum stress by downregulating the levels of transient receptor potential canonical (TRPC) 3 and TRPC6 (52). A significant decrease in tensile strength was also observed in DOX mice, while TSNIIA-SS treatment partially reversed the decrease in tensile strength (48).

### 3.4. Possible mechanisms

#### 3.4.1. Cells apoptosis and endoplasmic reticulum stress

Fourteen studies reported that the cardioprotective effect of AISM is related to apoptosis related pathways. After DOX treatment, the B-cell lymphoma 2/Bcl-2-associated × protein (Bcl-2/Bax) ratio in myocardial cells decreased, which promoted the process of cell apoptosis. However, pretreatment with Tan IIA reversed the DOX induced effect by inhibiting Bax expression, upregulating Bcl-2 levels, and restoring the Bcl-2/Bax ratio to normal (46, 56) Tan IIA also promoted the expression of B-cell lymphoma-extra-large (Bcl-xl), inhibited the increase of caspase-3 activity induced by DOX, reduced the release of cytochrome c, and downregulated the expression of cleaved Poly (ADP-ribose) polymerase (PARP). At the same time, protein kinase B (Akt) signaling pathway was also involved in the effect of Tan IIA on DOX induced cardiomyocyte apoptosis (49). The study also found that Tan IIA upregulated the expression of miR-133 and reduced cell apoptosis by inhibiting the expression of caspase-9 and related downstream signaling molecules (53). In addition, Tan IIA intervention increased the expression of phosphorylated extracellular signal-regulated protein kinase 1/2(p-ERK1/2), phosphorylated mitogen-activated protein kinase (p-MEK), and death domain-associated protein (DAXX) in myocardial cells, and decreased the expression of cleaved caspase-3, cleaved caspase-8, and p-P38, indicating that Tan IIA alleviated dox-induced cardiac cell apoptosis by activating the DAXX/MEK/ERK1/2 pathway (64).

Sal A treatment protected Dox induced cardiomyocyte apoptosis by inhibiting the activation of the nuclear factor-κB (NF-κB) signaling pathway and the expression of nuclear factor kappa B subunit 1 (NFKB1) (61). Sal B was also observed to have a similar effect. In the DOX group, the levels of caspase-3 and caspase-12 significantly increased, and the Bcl-2/Bax ratio decreased, but these changes were reversed by Sal B pretreatment. Glucose-regulated protein 78 (GRP78) and CCAAT-enhancer-binding protein homologous protein (CHOP) are markers of endoplasmic reticulum stress, and Sal B significantly reduces the expression of GRP78 and CHOP (50, 52). While, DOX treatment increased the expression level of endoplasmic reticulum related apoptosis proteins, including phosphorylated inositol requiring enzyme 1 (p-IRE-1), phosphorylated c-Jun N-terminal kinase (p-JNK), activating transcription factor-6 (ATF-6) and phosphorylated PKR-like ER kinase (p-PERK), while Sal B pretreatment inhibited these protein levels. Phosphatidylinositol 3-kinase (PI3K)/Akt is a survival regulation pathway, which saves cardiac systolic dysfunction by inhibiting endoplasmic reticulum stress. Sal B partially attenuated DOX induced endoplasmic reticulum stress by activating the PI3K/Akt signaling pathway, thus playing an anti-apoptotic role.

In another study, CPT treatment upregulated PI3 kinase p85 and p-AKT, inhibiting the expression of 14-3-3σ and p-JNK (58). CPT also regulated the levels of Bcl-2, Bax, Cleared caspase 3, and caspase 7, thereby alleviating DOX induced cardiomyocyte apoptosis (60). Moreover, the expression of cleared caspase 3 was increased in the left ventricle of mice treated with DOX. DHT I and DSS treatments both inhibited the expression of cleared caspase 3 and regulated the levels of Bcl-2 and Bax. Further research has shown that the anti-apoptotic effect of DHT I was partially mediated through the mammalian target of rapamycin (mTOR) pathway (59, 63). SMAE also blocked DOX induced cell apoptosis response by regulating ERK1/2 and p53 signaling pathways (57).

In addition, DB pretreatment increased the expression levels of Bcl-2, Bcl-xl, and survivin, and decreased the expression levels of Bax, p-p53, cytochrome c, and lysozyme 3, 7, 8, and 9. The protective effect mediated by it increases with the expression of c-Jun N-terminal kinase 1/2 (JNK1/2). Therefore, DB protects DOX induced cardiomyocyte apoptosis by activating the JNK1/2 pathway (55).

#### 3.4.2. Oxidant stress

Fifteen studies suggested that AISM exerted cardiac protective effect in different DIC models by decreasing oxidative stress. Tan IIA has significant antioxidant activity, which reduced DOX induced peroxide production in myocardial cells, inhibited the release of superoxide anion free radicals, and alleviated oxidative stress damage to mitochondria (44, 46, 49, 54). In the DIC model, Tan IIA pretreatment inhibited the production of ROS in a dose-dependent manner, reduced the production of malondialdehyde (MDA), and increased the activities of superoxide dismutase (SOD), catalase (CAT), and glutathione (GSH). Tan IIA pretreatment also induced the nuclear accumulation of Nrf2 and its downstream genes heme oxygenase-1 (HO-1), NAD(P)H dehydrogenase (quinone) 1 (NQO1), and glutamate-cysteine ligase catalytic subunit (GCLC) in both the mice cardiac tissues and H9c2 cells. This indicates that Nrf2-dependent antioxidant response mediates the protective effect of Tan IIA on DIC. Similarly, DSS can reduce the production of ROS, increase the levels of antioxidant enzymes such as SOD, CAT, and glutathione peroxidase (GSH-Px), and exert its effect by inhibiting the activation of the Keap1-Nrf2/NQO1 signaling pathway (63). Tan I has also been shown to enhance the expression of antioxidant enzymes such as HO-1 and NQO1 by activating the Nrf2 signaling pathway (63).

Tong Jun Lin et al. found that in the presence of ferrous ions, DOX stimulated the lipid peroxidation of mitochondria (43). The addition of Sai A could inhibit the formation of MDA in heart mitochondria induced by DOX, and in a dose-dependent manner eliminate the hydroxyl radical produced by DOX. Similar to DB (55), salvianolic acid exerts its antioxidant effect by inhibiting the accumulation of ROS (47). In addition, SMAE (45, 57), CPT (51, 58, 60), and DHT I (59) also inhibited DOX induced increase in myocardial MDA levels. The first two effectively eliminated ROS production and increased the content of antioxidant enzymes such as CAT, SOD, and GSH-Px, thereby protecting the heart from ROS damage.

#### 3.4.3. Inflammation

Two literature studies investigated the mechanism of action of DHT I and DSS in the treatment of cardiac inflammation. Xiaoping Wang et al. (59) found that DHT I exerts anti-inflammatory effects through multiple pathways. Firstly, DHT I treatment inhibited the recruitment of macrophages and the activation of M1 type macrophages, thereby reducing inflammatory responses. At the same time, it inhibited the activation of the NF-κB signaling pathway and reduced the activation of NF-κB and the expression of downstream inflammatory genes. Further experiments have confirmed that DHT I exerts its anti-inflammatory effect by regulating the mTOR TFEB-NF-κB signaling pathway, inhibiting the phosphorylation levels of mTOR and S6K, and promoting nuclear recruitment of transcription factor EB (TFEB). Jia-Ying Qi et al. found that DSS reduced the levels of TNF-α and IL-6 in heart tissue, reducing the cardiac inflammatory response caused by DOX. Based on these results, DHT I and DSS have shown potential anti-inflammatory effects in the treatment of cardiac inflammation, providing an important research basis for the development of new treatment pathways (63).

## 4. Discussion

### 4.1. Summary of evidence

**Figure 3:**
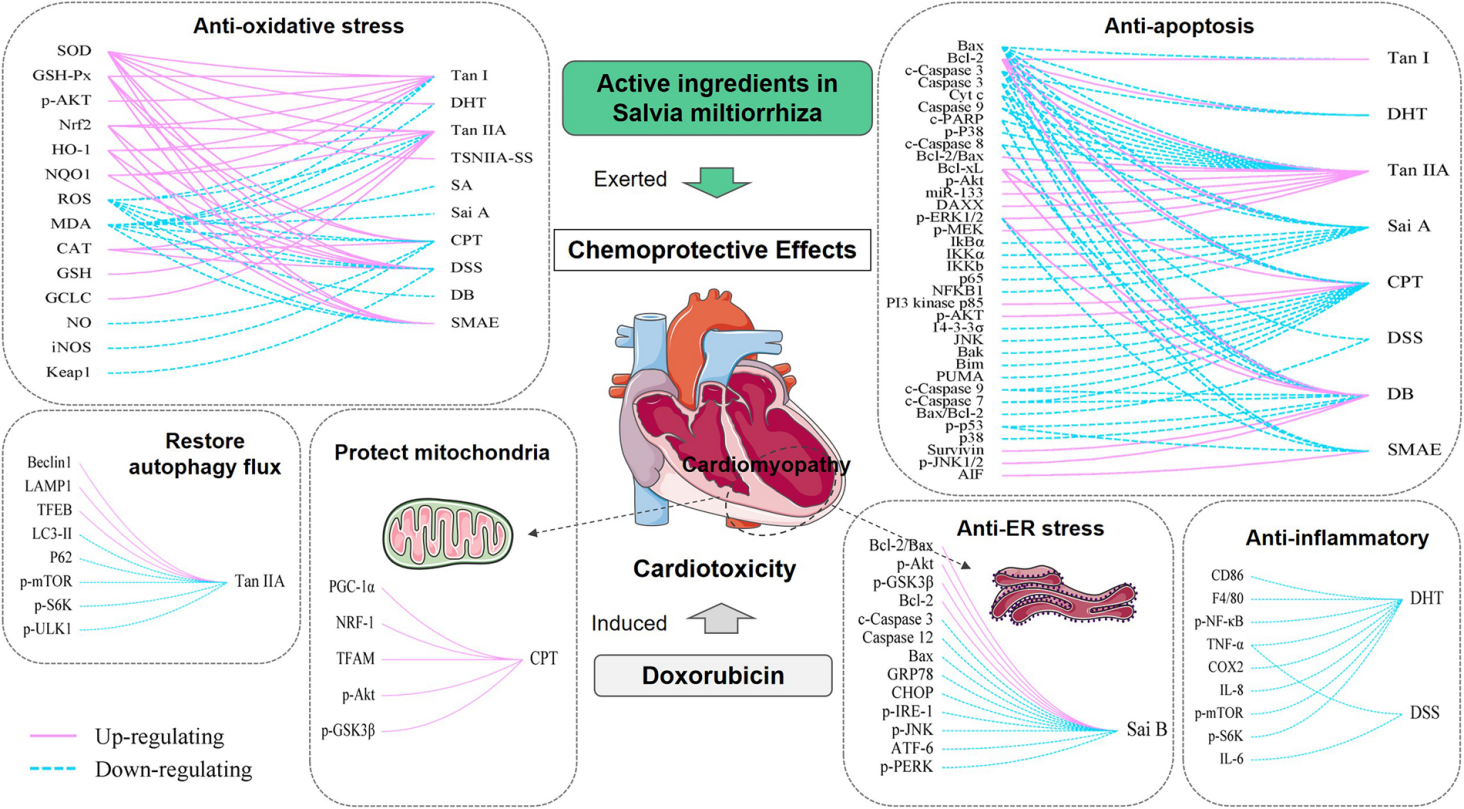
Mechanisms involved in AISM chemoprotective effects in DIC. Each box represents a function of AISM. On the right side of the curve are different types of AISM, and on the left side are the factors that are regulated by them. The solid pink line represents upregulation, and the dashed blue line represents downregulation. By regulating these factors, AISM can exert antioxidant stress, antiapoptotic, anti-endoplasmic reticulum stress, anti-inflammatory role, affect cell autophagy, and improve mitochondrial function, thereby exerting a cardioprotective effect on DIC. Tan I, Tanshinone I; DHT, Dihydrotanshinone I; Tan IIA, Tanshinone IIA; TSNIIA-SS, Tanshinone IIA sodium sulphonate; SA, Salvianolic acids; Sai A, Salvianolic acid A; Sai B, Salvianolic acid B; CPT, Cryptotanshinone; DSS, Danshensu; DB, Diethyl Blechnic; SMAE, *Salvia miltiorrhiza* aqueous extract.

Adriamycin is a widely used antineoplastic agent. However, the clinical use of adriamycin is limited by its unique cardiotoxicity. The ideal solution is to use natural chemical protectants during DOX treatment to reduce adverse reactions and improve patients' survival rates. Our study summarizes the protective effect of active ingredients in *Salvia miltiorrhiza*, including Tan I, DHT I, Tan IIA, TSNIIA-SS, SA, Sai B, CPT, DSS, DB, and SAME, which were described in the literature as cardioprotective agents. The functions include: improvement of physical signs and biochemical indicators, protection of cardiac function damage caused by DOX, alleviation of the development of cardiac toxicity, reduction of myocardial lesions and protection of heart tissue structure, enhancement of myocardial cell viability, prevention of cardiomyocyte apoptosis, increase of the chemosensitivity of cancer cells to DOX, *etc*. They also exerted myocardial protective effects by protecting the integrity and function of mitochondrial membranes, promoting mitochondrial biogenesis. The cardioprotective effect of AISM involves a variety of mechanisms that are related to inhibiting apoptosis, decreasing inflammation, attenuating oxidative stress, suppressing endoplasmic reticulum stress, affecting cellular autophagy and calcium homeostasis (Figure 3). These results provide sufficient evidence for further clinical studies.

### 4.2. Implications for future directions and clinical practice

We reviewed the literature on the cardioprotective effects of AISM on DOX induced cardiotoxicity, providing clinicians with options to address cardiotoxicity through different avenues. Some extracts were more concerned by researchers than other phytochemistry substances, such as tanshinones, salvianolic acids, etc. Many of these compounds have multiple mechanisms of action, including antioxidant stress, anti-apoptotic activity, as well as anti-inflammatory effects. However, considering the dosage range and treatment time of AISM and DOX, as well as the frequency of administration and the starting time, the diversity of research designs makes it difficult to compare different studies and draw reliable conclusions. These limitations also hinder the comparison of AISM with each other to select a compound with higher potential. In addition, most studies mainly focused on DOX induced cardiotoxicity, with only a few studies targeting organs such as the liver, kidneys, brain, or testicles that are also affected by DOX. Therefore, future research should aim to clarify and demonstrate the administration plans of different compounds, as well as clarify the toxicity of DOX and the effects of *Salvia miltiorrhiza* compounds on these organs (65).

At present, the evidence on the cardioprotective effect of *Salvia miltiorrhiza* compounds is mainly limited to preclinical trials. Unfortunately, our literature search did not find any clinical trials in this regard. The reasons for the lack of clinical trials are multiple, like other phytochemistry substances, which may include several aspects: lack of evidence on the bioavailability of these compounds in humans; Most of these studies were conducted on cells or animal models lacking cancer cells; It is unclear whether these compounds affect the anti-tumor activity of doxorubicin. A common concern is whether the use of antioxidants will interfere with the effects of chemotherapy drugs by preventing ROS damage to cancer cells (66). An ideal cardioprotective agent should not only prevent cardiotoxicity, but also should not interfere with the required action of DOX. Therefore, it is recommended that future research focus on developing synthetic derivatives of these compounds to enhance their bioavailability in human tissues; Future research should be recommended to use cells or animal models containing cancer to test the possible interactions between these compounds and DOX anti-tumor activity; Design high-quality clinical trials to validate the findings observed in preclinical models (67).

Although *Salvia miltiorrhiza* compounds have significant protective effects, their adverse pharmacokinetic/pharmacodynamic characteristics limit their application. The clinical application of tanshinone IIA in anti-cancer treatment is hindered by its low water solubility, low cell uptake, short half-life, and first-pass metabolism (68–70). Therefore, in the future, attention needs to be focused on optimizing delivery strategies for these chemical protectants to overcome these limitations. Currently, some research results have been reported. Guanxing Sun et al. constructed a drug delivery system for the co-delivery of DOX and TAN. Lipid nanoparticles loaded with DOX and TAN (N-DOX/TAN) were prepared by emulsification and solvent-diffusion methods. Prostate-specific membrane antigen (PSMA) targeted nanoparticles loaded with DOX and TAN were synthesized by conjugating a PSMA targeted ligand to N-DOX/TAN. Through *in vitro* and *in vivo* experiments, it has been found that the novel nanomedicine offers great promise for the dual drug delivery to prostate cancer cells, showing the potential of synergistic combination therapy for prostate cancer (71). These new delivery systems have been reported to improve the bioavailability of AISM, promote their protective effects on the heart and kidneys, and more importantly, maintain the anticancer and anti-tumor efficacy of DOX (72–75). It should be noted that the data represented in the current system review is based on *in vitro* and *in vivo* models. Therefore, in the future, it is recommended to use *Salvia miltiorrhiza* compounds as chemical protectants in combination with doxorubicin for cancer patients, which requires further research, as sometimes the results of *in vitro* and *in vivo* models and clinical studies may differ.

### 4.3. Limitations

Some limitations should be addressed. Firstly, studies evaluated in this systematic review were inconsistent in some important aspects, including the types of AISM, durations, dosages, and routes of administration of AISM and DOX, which reinforced the heterogeneity of the studies. Thus, meta-analyses were not performed for the accessed data. Secondly, according to the SYRCLE's tool, many of the studies suffer from risk of bias. Most studies only mentioned “randomization”, but did not introduce specific approaches. All of the studies received an “NC” rating for the allocation of concealment items and the random outcome assessment. Thirdly, the review was limited to studies published in English only, so there are language and regional differences, literature published in other languages may be missed.

## 5. Conclusion

The findings showed that doxorubicin chemotherapeutic agent can induce the changes in biochemistry and histology of the cardiac cells/tissue. However, using active ingredients in *Salvia miltiorrhiza* alleviate the doxorubicin-induced adverse effects, does not affect or even enhance the anticancer effect of DOX. Mechanically, active ingredients in *Salvia miltiorrhiza* exert their chemoprotective effects through several main mechanisms of antiapoptosis, antioxidant, anti-ER stress, and anti-inflammatory.

## Data Availability

The original contributions presented in the study are included in the article/**Supplementary Material**, further inquiries can be directed to the corresponding author.

## Glossary

AIF, apoptosis-inducing factor; Akt, protein kinase B; ANT, adenine nucleotide translocator; AST, aspartate transaminase; ATF-6, activating transcription factor-6; Bak, Bcl-2- antagonist/killer; Bax, Bcl-2-associated × protein; Bcl-2, B-cell lymphoma 2; Bcl-x_L_, B-cell lymphoma-extra-large; Bim, Bcl-2 interacting mediator of cell death; CAT, catalase; CHOP, CCAAT-enhancer-binding protein homologous protein; CK, creatine kinase; CK-MB, creatine kinase-muscle/brain; COX2, Cyclooxygenase-2; CPT, cryptotanshinone; Cyp-D, cyclophilin D; Cyt c, cytochrome c; DAXX, death domain-associated protein; DHT, dihydrotanshinone; DIC, doxorubicin-induced cardiotoxicity; DOX, Doxorubicin; DSS, danshensu; ECG, electrocardiography; EF, ejection fraction; ERK1/2, external-signal regulated kinase1/2; FS, fractional shortening; GCLC, glutamate-cysteine ligase catalytic subunit; GRP78, glucose-regulated protein 78; GSH, glutathione; GSH-Px, GPX, glutathione peroxidase; GSK3β, glycogen synthase kinase 3β; HO-1, heme oxygenase-1; HW/TL, heart weight/tibia length; IkBα, NF-kappa-B inhibitor alpha; IKKα, IkB kinase-α; IKKβ, IkB kinase-β; IL-8, interleukin 8; iNOS, inducible nitric oxide synthase; JNK, p-c-Jun N-terminal kinase; JNK1/2, c-Jun N-terminal kinase 1/2; Keap1, Kelch-like ECH-associated protein 1; LAMP1, lysosomal-associated membrane proteins-1; LC3-II, light chain 3-II; LDH, lactate dehydrogenase; LVEDD, left ventricular end-diastolic dimension; LVEF, left ventricular ejection fraction; LVESD, left ventricular end-systolic dimension; LVFS, left ventricular fractional shortening; LVIDd, left ventricular internal diameter at diastolic phase; LVIDs, left ventricle internal diameter in systolic phase; MDA, malondialdehyde; MMP, mitochondrial membrane potential; MRP2, multidrug resistance-associated protein 2; mTOR, mammalian target of rapamycin; NFKB1, nuclear factor kappa B subunit 1; NO, oxidative stress-relative enzymes nitric oxide; NQO1, NAD(P)H dehydrogenase (quinone) 1; NRF-1, nuclear respiratory factor-1; Nrf2, nuclear factor (erythroid-derived 2)-like 2; PARP, Poly (ADP-ribose) polymerase; PGC-1α, Peroxisome proliferator-activated receptor γ coactivator-1α; P-gp, P-glycoprotein; PI3K, phosphatidylinositol 3-kinase; p-IRE-1, phosphorylated inositol requiring enzyme 1; P-JNK, phosphorylated c-Jun N-terminal kinase; p-MEK, phosphorylated mitogen-activated protein kinase; p-NF-κB, phosphorylated nuclear factor-κB; p-PERK, phosphorylated PKR-like ER kinase; PUMA, P53 up-regulated modulator of apoptosis; PV, pressure-volume; ROS, reactive oxygen species; Sai A, Sai B, salvianolic acid A and B; AISM, active ingredients in *Salvia miltiorrhiza*; SOD, superoxide dismutase; Tan I, tanshinone I; Tan IIA, tanshinone IIA; Tan IIB, tanshinone IIB; TBARS, TBA reactive substrate; TFAM, Transcription Factor A, Mitochondrial; TFEB, transcription factor EB; TNF-α, tumor necrosis factor-α; TRPC, transient receptor potential canonical; T-SOD, total superoxide dismutase; ULK1, UNC-51-like kinase 1
