# Supplementary material for: Potential chemoprotective effects of active ingredients in Salvia miltiorrhiza on doxorubicin-induced cardiotoxicity: a systematic review of in vitro and in vivo studies
Source: Front Cardiovasc Med. 2023 Oct 17;10:1267525. doi: 10.3389/fcvm.2023.1267525 (PMC10616797; doi:10.3389/fcvm.2023.1267525)
Supplement: Supplementary file 1 [file Datasheet1.pdf]

Potential Chemoprotective Effects of Active ingredients in Salvia miltiorrhiza on Doxorubicin-Induced Cardiotoxicity: A Systematic Review of *In vitro* and *In vivo* Studies

Pubmed
(Doxorubicin) AND ((((((Salvia miltiorrhiza) OR (Danshen extract)) OR (tanshinone)) OR (Cryptotanshinone)) OR (Danshensu)) OR (Salvianolic acid))

WOS
Salvia miltiorrhiza (All fields) or Danshen extract (All fields) or tanshinone (All fields) or Cryptotanshinone (All fields) or Danshensu (All fields) or Salvianolic acid (All fields)
AND
ALL=(Doxorubicin)

Embase
'salvia miltiorrhiza':ab,ti OR 'danshen extract':ab,ti OR tanshinone:ab,ti OR cryptotanshinone:ab,ti OR danshensu:ab,ti OR 'salvianolic acid':ab,ti
AND
doxorubicin:ab,ti

Scopus
((TITLE-ABS-KEY(Salvia miltiorrhiza) OR TITLE-ABS-KEY(Danshen extract) OR TITLE-ABS-KEY(tanshinone) OR TITLE-ABS-KEY(Cryptotanshinone) OR TITLE-ABS-KEY(Danshensu) OR TITLE-ABS-KEY(Salvianolic acid))) AND (TITLE-ABS-KEY(Doxorubicin))

Cochrane
#1: Salvia miltiorrhiza OR Danshen extract OR tanshinone OR Cryptotanshinone OR Danshensu OR Salvianolic acid
#2: Doxorubicin
#1 AND #2
